# A blockchain-empowered AutoML reputation system for anonymous customer service in SMEs

**DOI:** 10.3389/frai.2026.1846713

**Published:** 2026-08-17

**Authors:** Selvalakshmi Annamalai, Logesh Ravi

**Affiliations:** 1 School of Computer Science and Engineering, Vellore Institute of Technology, Chennai, India; 2 Centre for Advanced Data Science, Vellore Institute of Technology, Chennai, India; 3 School of Electronics Engineering, Vellore Institute of Technology, Chennai, India

**Keywords:** AutoML, blockchain, customer services, reputation system, small and medium-sized enterprises

## Abstract

A typical manual customer service model for small and medium-sized enterprises (SMEs) is transitioning to automated customer service, using various computing resources to deliver more effective and high-quality services. The prerequisites for automated customer service include extensive data and strong data analytics capabilities. Unfortunately, because of their small size and lack of resources, many SMEs, particularly small and medium-sized companies, lack adequate data and ability. SMEs are forced to rely on external computing sources, which prevents them from developing core competencies in customer service. To address this issue, we propose a blockchain-enabled and automated machine learning (AutoML) model that uses decentralized datasets for customer services. Blockchain, a secure and transparent digital ledger, ensures the integrity and traceability of data, making it an ideal solution for storing and managing customer service data. Blockchain smart contracts allow customer services to be carried out automatically in a pipeline, producing review scoring reputation systems. The proposed scheme includes the following: model implementation, enterprise registration, review aggregation, verification, and review evaluation integrated into blockchain. To guarantee that the customer service system is traceable and that the data of each reviewing enterprise is safely, effectively, and impenetrably stored on the blockchain nodes, we first present a mechanism for review data administration and storage utilizing blockchain. Next, we provide a pipeline that efficiently integrates the crucial processes of model creation, review collection, verification, and model assessment using an AutoML model. The experimental findings show that our proposed system reliably and effectively assesses customer service in SMEs.

## 1 Introduction

In recent times, SMEs have been affected by cybersecurity much more than in the past. When it comes to protecting their data, SMEs must strike a compromise. Standard precautions are being implemented through legal changes, Brexit, UK GDPR, and cyber essentials (Saleem, 2019), among many other initiatives, because of the rise in cyberattacks brought on by the proliferation of innovative technology. Helping SMEs grasp the more abstract ideas behind AI and ML may be beneficial (Khan et al., 2023). Establishing these standards involves controlling the many connections and interactions made on the internet and intervening in current cybersecurity precautions. Using ML methodologies is one developing possibility that enables organizations to determine the cause-and-effect links between breaches and their effects on SMEs. Utilizing statistical methods exposes distinctive patterns and behaviors for zero-day cyberattacks. The relationships among various anomalous factors may enhance the safety of data management in the SME context. Apart from the different technologies and organizations contributing to the multitude of variables changing cybersecurity, human factors also play a significant role in the security of the Internet of Things (IoTs) and devices. Several technical advisory organizations provide online resources to help SMEs comprehend and coexist with IoTs. Technology, privacy, security, and legal difficulties are further worsened by anti-forensic techniques, authority, and service level agreements (SLAs). The GDPR and human aspects associated with IoTs enable the safety and security of IoTs in an ecosystem of SMEs.

Technology usage has also changed because of Industry 4.0. It is not surprising that firms and SMEs found it difficult to make judgments in the pre-pandemic era and base their decisions on their best operations (Kumar et al., 2023). Making the most of AI and ML would be necessary to enhance internal processes and capacities, including company finance management and operations. The International Institute for Management Development (IMD) considered that AI worked best when there was a historical database to draw from and use to make more correct predictions. This is true because there is a better prediction chart to display the “best fit” line when more historical data, or Big Data, is gathered. Recent research papers continue by discussing data visualization; in the past, companies could have looked at complicated events and business drivers to propel their enterprise ahead when, from a fundamental perspective, success was possible. The IMD discussed the epidemic that led to the creation of a more basic model that people might use to live. Demand and supply changed, and its platform and population migrated to cyberspace, a more robust virtual world devoid of COVID-19 (Jabeen et al., 2023). People’s awareness is in jeopardy, given that humans need to get to the next level of design using machine learning and begin engaging with computers more quickly.

In recent decades, data has emerged as a crucial source of information and, through clever applications, has opened new avenues for solving issues in real-world industries, including banking, bioinformatics, agriculture, and wireless communications (Hongyun et al., 2023). These data-driven applications integrate actionable information into the user interface, allowing people to carry out intended activities more quickly. This makes insights operational, enhances customer interactions, personalizes the customer experience, boosts operational effectiveness, and opens new business opportunities. Various intelligent applications, such as smart grids, UAVs, and smart cities, can simplify human life. These apps produce enormous amounts of data, which present challenges for database storage and interaction due to their constantly changing nature.

Blockchain technology, featuring a distributed network, can address these problems. It was created in 2008 by Satoshi Nakamoto and involves a collection of networked computers that work together to keep time-stamped, tamper-proof records (Nakamoto, 2008). It holds a series of blocks joined by cryptographic building blocks. Immutability, decentralization, and openness are the three pillars of blockchain. These three features have enabled various applications, such as digital currency (currency that does not exist physically) and the study of its appropriateness for intelligent application development. While blockchain technology guarantees privacy and security, several weaknesses surfaced after its implementation. For example, the nature of assaults started getting more complex, such as majority attacks (51%) that control voting and Sybil attacks that generate phony identities to manipulate consensus.

**Figure 1:**
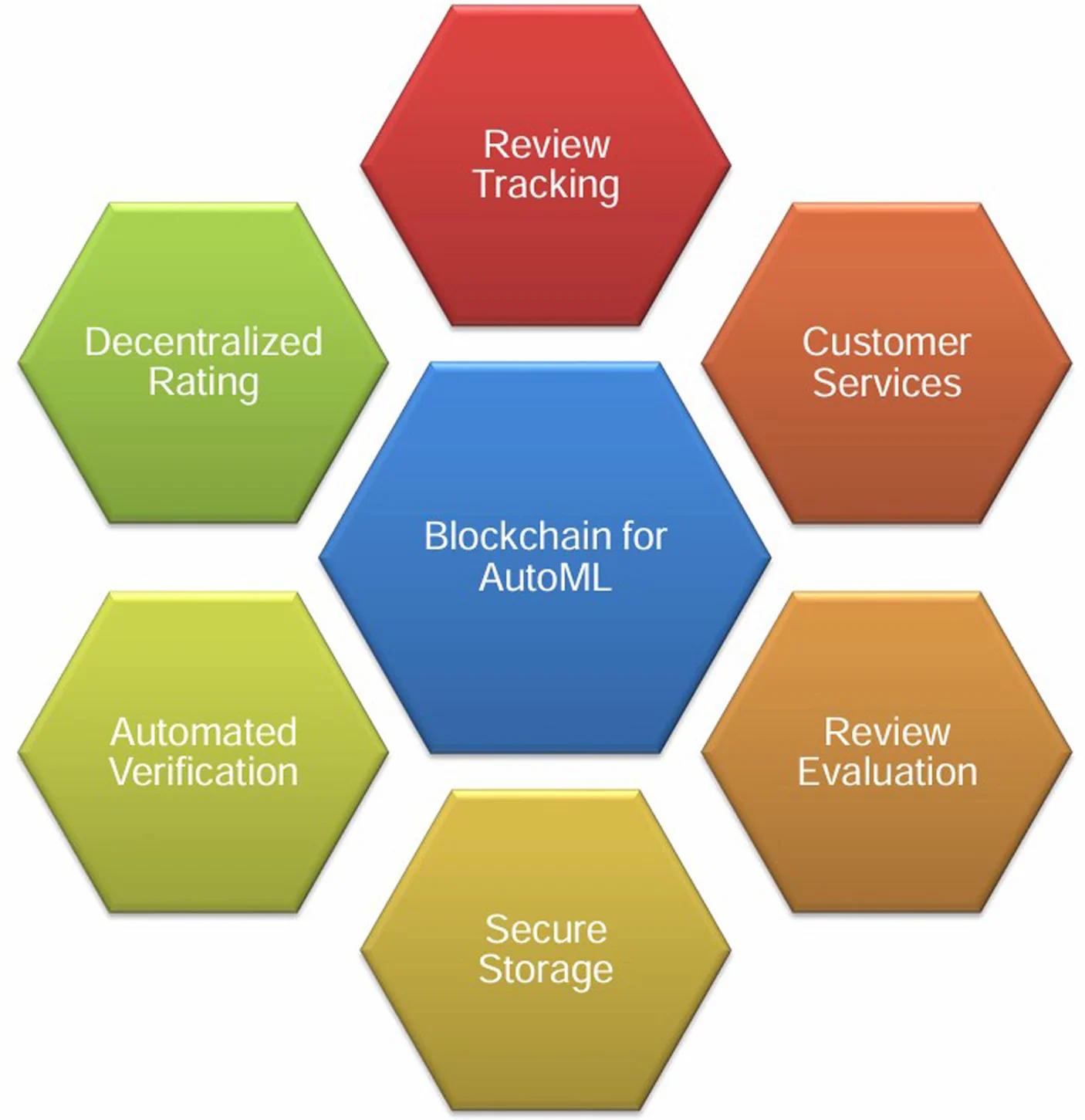
Blockchain for AutoML applications.

Since old approaches rely on signatures to find patterns, a robust intrusion detection system (IDS) is necessary to address the previously described problem (Tirumala et al., 2022). However, machine learning (ML), one of the newest technologies, may be used to analyze data flow and find patterns of intrusions and attacks. Therefore, creating reliable and effective methods to evaluate this enormous volume of data is essential for managing blockchain-based intelligent systems (Sasikumar et al., 2023). Trust management is one of the crucial components for a decentralized network to enhance the operation of digital twins and effectively manage data within the network. As a result, machine learning is widely used today, often without the user even realizing it. ML allows computers to learn, think, and act without human involvement. This is seen as one of the uses for AI. ML enables computers to learn without explicit programming. Its main idea is to create a practical algorithm to take in data, make a forecast using statistical analysis, and change its results. ML can analyze a large quantity of data to provide choices based on facts. The benefits of integrating blockchain with AutoML are shown in Figure 1.

The retail business is radically transforming because of Industrial IoT (IIoT), a worldwide network of intelligent things. Suppliers, manufacturers, and retailers are implementing IIoT in a global retail ecosystem to lower supply chain management costs and enhance manufacturing operational efficiency. IIoT is also expected to help retail marketing by using Big Data and cloud computing technology to address the demands of globalization, competitive markets, and diversified customer demand. Retailers can better manage their businesses by gathering vast amounts of feedback from multiple sources and devices using IIoT technologies. Retailers depend heavily on customer feedback to develop their brands with business associates and win over new customers (Nylander et al., 2017).

Customers can provide feedback on their interactions with shops (often through a review message or a rating score). These comments build up over time and might be listed by other retail sector organizations. Still, a few complex issues can prevent the creation of a trustworthy retail reputation system. Initially, providing feedback can reveal a great deal of personal consumer data that can be used to watch and categorize customers (Suh and Han, 2003). Additionally, customers may feel pressured or unwilling to give a particular shop a poor review out of concern for the implications. This issue, which may be subject to de-anonymization assaults, cannot be resolved by relying solely on pseudonyms to provide rating anonymity (Hoyer and van Straaten, 2022). Second, most reputation systems today rely on a centralized marketplace to gather and compile user evaluations. Retailers can more effectively run their businesses by collecting vast amounts of feedback from multiple sources and devices using IIoT technologies. Retailers depend heavily on customer feedback to develop their brands with business associates and win over new customers (Nguyen and Simkin, 2017). Customers are expressly allowed to provide feedback on their interactions with businesses, typically as a review message or a rating score. These comments build up over time and might be listed by other retail sector SMEs (Li et al., 2021). However, it has been proved that the need for system openness and the leakage of private customer information may lead the existing centralized marketplace to fall short of its promise of the required degree of confidence (Yang et al., 2023). Research has been done on creating a reputation structure that does not rely on a centralized market and offers robust customer privacy assurance (Soska et al., 2016). In addition to supporting anonymity, reputation networks must withstand several assaults, which become increasingly difficult in a decentralized economy. Due to the absence of mutual trust between the relevant stakeholders, decentralized reputation system approaches do not offer enough system transparency, which is crucial for IIoT-enabled commerce marketing. Much study has gone into creating a blockchain-based structure to achieve a more open and forthright reputation structure.

As a result, outsourcing automated customer services by today’s SMEs (Selamat and Windasari, 2021) raises review and verification issues. Certain SMEs give their customers’ data to data analytics firms, who then provide the SMEs with the findings of their analysis. This procedure, when conducting data transactions, creates a trustworthy system for data authenticity, which carries a risk of disclosing consumer information to third parties. To ensure that data analytics businesses do not use the information for other goals, it is also necessary to set up a very trustworthy connection with them. Moreover, companies could be unable to decide the choice or outcome. As a result, they cannot learn and improve their core ability in customer service. Controlling and guaranteeing the quality of client services is another challenge.

To address the above-mentioned issues, the article proposes an automated customer support infrastructure based on blockchain and AutoML for SMEs. A chain of data created utilizing cryptographic connections makes up the decentralized database known as blockchain. Many network connections properly confirm each data point in the string. Blockchain combines several technological methods, such as information encryption, peer-to-peer transfer, consensus systems, and distributed and decentralized data storage. In distributed systems, decentralized data storage and transactions are realized when a trustworthy intermediary is unnecessary. Blockchain ensures that existing data cannot be altered by using cryptography. It employs a consensus method to agree on fresh data among several nodes. Coordination and cooperation address trust, dependability, and security issues that often arise with centralized approaches. As a result, blockchain technology may guarantee that data is not altered and that a trustworthy setting free from centralized management is offered. Combining blockchain technology with AutoML enhances the SMEs’ operational ability by introducing an automated customer service system. The trust of the customer review system is stored in the blockchain to provide traceability of the proposed system.

### 1.1 Contributions

As noted, creating machine learning models is an endeavor that is intensive in ability and time. AutoML aims to automate these procedures so non-expert users can develop machine learning models. This study proposes decentralized AutoML, which combines blockchain, IoT, and AutoML to enable parties to exchange massive customer data and models (Karmaker et al., 2021). Leveraging each other’s efforts can lower the implementation costs of automated customer care.

This work makes three primary contributions:

1. This work introduces an innovative combination of blockchain and AutoML. This novel approach could revolutionize the way SMEs manage their customer service, leading to automated customer service in SME organizations.
2. The proposed platform offers significant advantages for SMEs. By harnessing large volumes of collective consumer data for continuous customer service improvement, it can level the playing field for SMEs, particularly those with limited resources and size, giving them a competitive edge in the market.
3. The viability of the suggested strategy is evaluated by contrasting the outcomes with those of various blockchain technologies. The simulation outcomes show that the proposed method has minimal latency and can handle more data.

The rest of the article is structured as follows: Section 2 presents the related works. Section 3 provides the system model, security model, and design flows. Section 4 has the analysis of the proposed architecture, and we examine the security of the proposed model. Section 5 concludes the paper.

## 2 AutoML and Blockchain in SMEs

### 2.1 Blockchain technology

Blockchain technology is used in this work to improve SME ecosystem protection. A blockchain is a distributed, unchangeable database where newly added time-stamped transactions are bundled and linked to the block hash-chain. The core blockchain protocols decide the number of replicated copies of the block that may be produced and kept alive. Deciding how a network of users, referred to as miners, might reach a consensus on the current state of the blockchain is a critical part of this system. There are four types of blockchain architecture: permissioned, permission-less, private, and public. There are two popular methods for doing the same, including Proof of Work (PoW) and Proof of Stake (PoS) (Wendl et al., 2023). The new transaction is added to the blockchain after this process is finished. Every block holds a code called a hash connecting the blocks in a specific sequence. Hashes are composed of the block’s hash before it in the chain. A miner should use a series of calculations to build their leadership credibility. This calculation solves the problem of translating data from variable sizes to fixed sizes. One approach might be to choose a leader in other networks. Several miners try to solve the challenge in PoW, with the miner who finishes first broadcasting to the group proof that the task is completed. Ten other miners confirm the accuracy of the job they have conducted. They all choose that miner as the leader once they have confirmed this. Maintaining a list of verified transactions using a cryptographic hash function is the primary goal of the block. The following features make the hash function efficient (Abed et al., 2021).

- Regardless of the length of the input, it generates an output with a defined length.
- It is deterministic, producing comparable results given an input.
- It is irreversible, making it impossible to obtain a comparable input from the output.
- New output is produced whenever the original input is altered.
- There is less overhead and quicker hash computation.

The hashes confirm the block on the blockchain, which is linked to the original genesis block. Every block is connected using the connection between each hash, showing that every block contains the earlier hash and is later hashed in the next block. Since the first hash is linked to the following block in the chain, any alteration to the hash breaks the chain. Recalculating the original hash is necessary to restore the chain and takes significant processing power. The nonce is also included, so the miner manipulates the data to produce a hash that outputs three zeroes. The block is eventually considered genuine and published to the network when the miner discovers a nonce that causes the block hash to fall below the strict threshold.

A smart contract is a program or collection of scripts that runs on the blockchain and adds new blocks in response to specific events. It converts actual legal contracts into blockchain-enforcement programs (Khan et al., 2021). It is comparable to conventional databases’ stored processes. Smart contracts are kept as scripts on the blockchain to generate the intended results from the first contract and performed based on the provided data. It controls transactions conducted as a whole or part, depending on the input. A smart contract’s primary goal is to offer better security while reducing expenses and waiting times connected with regular contracts. Blockchain can be used in customer services as it can provide process transparency and preserve the integrity of data storage. The absence of universal trust suggests that blockchain networks require a distributed consensus method for block validation.

### 2.2 AutoML

In artificial intelligence (AI), ML models employ algorithms to acquire knowledge from data to find patterns and new information. Significant accomplishments have been made because Big Data is now readily available and has many uses. In real-time, creating machine learning models is expensive and time-consuming, and SMEs need help. Specifically, AutoML looks to automate costly and time-consuming procedures based on transfer learning, hyperparameter, and stochastic optimization. Learning algorithms’ hyperparameters can be changed via optimization techniques. Integrating several characteristics of ML algorithms enhances the efficiency of stochastic optimization models. By moving learning parameters and data from a single program to a different one, transfer learning is used to speed up model configuration. It is beneficial for recently developed applications with a small amount of real-time data. AutoML technology can quickly handle massive volumes of customer service data, which helps manage customer service data’s volume, diversity, and velocity (He et al., 2021). Therefore, combining blockchain technology with AutoML seems like a practical way to achieve automated customer support at a reasonable price.

AutoML methods are growing in popularity and are often used to solve real-world problems with regression and classification models. Using an AutoML-based dimensionality reduction technique, authors created an efficient machine learning classification capable of distinguishing COVID-19 instances from non-COVID-19 cases with good sensitivity and precision (Zargari Khuzani et al., 2021). Grid GRACE-like data restoration was conducted using an automated AutoML technique. Using numerous sets of meteorological and climatic data as predictors and six distinct machine learning scenarios, they proved the procedure over the continental United States. In (Ikemura et al., 2021), the authors used AutoML to train several machine learning methods. The approach that most accurately forecasted patients’ odds of sustaining an infection with SARS-CoV-2 was selected. The findings of this study underscore the practical implications of AutoML in enhancing the accuracy of models.

A neural network model-building process known as mAML was developed to reproducibly construct optimal and understandable models for personalized microbiome-based classification tasks (Yang and Zou, 2020). Thirteen benchmark datasets, encompassing binary and multi-class classification tasks, were used to prove the pipeline’s robust performance. This underscores how automated machine learning can significantly improve the effectiveness of creating machine learning models, thereby positively affecting the accuracy of classification or regression models. The study also highlights the progressive use of machine learning algorithms in credit scoring, with promising prediction findings.

Each machine learning component needs to be better incorporated, and most of the current research is still concentrated on the classification model itself. Furthermore, many non-specialists need to use the models for credit scoring quickly and accurately. This is challenging since the model involves an extensive understanding of data science concepts such as feature selection and hyperparameter tuning. As a result, the primary shortcomings of the current research on credit scoring are as follows: (1) The models must be built and adjusted by humans, necessitating a great deal of human action; (2) the study on credit scoring seldom considers feature engineering, thus missing the crucial step of choosing the appropriate features; and (3) the present techniques are distinct for each component, needing human manipulation and processing of the data among the various modules, thereby reducing the method’s integrity. Our suggested blockchain solution uses an automated machine learning strategy to allow data to flow throughout the process to overcome these problems. Reputation scoring systems significantly reduce the amount of human involvement by systematically processing the data by extracting features, selecting features, modeling, parameter choice, and model assessment.

### 2.3 Small and medium-sized enterprises

The implementation of ML in the small and medium-sized enterprise (SME) sector has many benefits, but there are also drawbacks. These include the lack of dataset accessibility for evaluation, the possibility of data skewing, and the requirement for data truthfulness (Zhu et al., 2017). This is because human involvement is still necessary to create models using data and arithmetic, and the extent of human-caused errors still needs to be defined. There has been much human interference thus far. Because of their ecology, SMEs are now particularly susceptible to intrusions. One explanation for this is the deficiency in cybersecurity resources and capabilities within the SME organizational structure. SMEs are increasingly being exploited, and the risk of cyberattacks comes at a heavy cost in terms of data breaches. SMEs need help keeping up with the increased risks associated with cybersecurity. For the least electronically developed SMEs, instinctive, threat-based internet risk evaluation approaches are used, which employ a socio-technical cyber safety structure to help meet their needs.

In summary, SMEs have the internal knowledge to build confidence in cybersecurity and possess regulations and processes. Digitally oriented SMEs and digital facilitators were recommended to use a more thorough risk evaluation strategy and competence model. Since digital enablers had the data and cybersecurity knowledge needed to make these solutions work, they were also excellent candidates for using more sophisticated aggregation techniques, including Bayesian networks (Wang and Chen, 2021). Threat-based risk evaluation, based on non-aggregated or natural methods, focuses on real-life dangerous surroundings that support sensations of competence and connection through guaranteeing best organization. The obstacles presented show that, unlike more prominent organizations, SMEs cannot use a “cut and paste” approach to understanding cybersecurity and its hazards.

Large Australian corporations have long been early adopters of cybersecurity solutions, often having the workforce, resources, and setting necessary to advance knowledge about cyber risks. According to (Gale et al., 2022), most cybersecurity conventions and lessons arose from early, significant catastrophes that primarily affected large organizations, such as NotPetya, Equifax, Wikileaks, etc. As such, larger organizations’ requirements impact industry best practices, standards, and products related to cybersecurity. The Technology Acceptance Model (TAM) further emphasizes that deploying solutions designed for more prominent organizations to smaller consumers is impossible since an SME’s technological environment may differ significantly from that of a large enterprise. Using the UK as an example, consider the introduction of GDPR and Cybersecurity (Stalla-Bourdillon et al., 2018). SMEs could afford the labor and technical resources to adopt more quickly, while larger organizations could take a more straightforward approach to implementation than SMEs. The technological architecture of small business IT also presents a challenge to implementing a comprehensive cybersecurity solution. TAM continues by saying that the requirement for a stable testing setting is still another significant obstacle to technical execution. In the context of this Australian example, testing settings among larger organizations may be achieved more easily than among SMEs. TAM investigates the need for a secure testing setting for any cybersecurity system to evaluate a reaction to catastrophic occurrences.

A denial of service (DoS) simulator can mimic an overloaded service, preventing valid requests from being processed. If a DoS simulator were deployed on a live system, it might compromise the entire system’s integrity and result in commercial loss for SMEs by making their IT infrastructure, such as websites, inaccessible to consumers (Alosaimi et al., 2016). TAM concludes that stressful exams cannot be administered in real-world settings. As a result, companies’ incident response training will never include testing the entire range of catastrophic situations if they do not have a test environment. TAM continues by discussing the significance of a test environment, which calls for high technical ability, effort, and continuous upkeep. These requirements are only realistic in more prominent companies and are seldom ever seen in the context of SMEs.

TAM’s study emphasizes impediments other than technological ones, such as psychological barriers, which make it difficult for SMEs to adopt the best cyber security decisions and advance the organizational and procedural maturity of the SME sector (Awa et al., 2015). Moving forward to safeguard their data has often been difficult for SMEs in Australia due to the complicated nature of applying industry standards and the associated expenses of cyber security coverage, legal remedies, and data breaches. TAM’s research fits nicely with the current technological environment comparable to that of the UK and will thus raise similar cyber security issues. Because SMEs in Australia and the UK have comparable social backgrounds, their difficulties with cyber security are identical. To aid SMEs in strengthening their defenses against cyberattacks, TAM’s study concludes that opportunities to apply non-traditional solutions to cyber safety have grown obvious through newly formed partnerships, security paradigms, and the community of open-source software. The literature analysis above proves that IoT, AutoML, and blockchain all have specific benefits when enhancing customer services. Integrating them to provide transparent and automated client support seems interesting. Nevertheless, studies on the fusion of these advancements are scarce.

### 2.4 Limitations and challenges of AutoML-integrated blockchain in SMEs

AutoML-integrated blockchain frameworks, by utilizing different ML models for data analysis, are employed to assist SMEs in overcoming technological obstacles. However, substantial computing costs, significant networking and scalability issues, and a general lack of internal knowledge impede its adoption for SMEs. Many SMEs are unable to build key competences internally because they must rely on expensive outside consultants or cloud providers for upkeep of their customer services. Reliability, openness, and traceability are some of the main issues with traditional customer services. Blockchain technology strengthens SME customer service and addresses these flaws. If the implementation issues are resolved, blockchain technology is fundamentally beneficial and functional. To overcome these challenges and limitations, in this work we have proposed an AutoML-integrated reputation system based blockchain framework for SMEs customer services. The main aim of this work is to provide reliable services to customer reviews based on the reputation scoring mechanism. The following section details the proposed framework FOR automated customer services in SMEs.

## 3 Proposed architecture of the blockchain-integrated AutoML for SMEs

**Figure 2:**
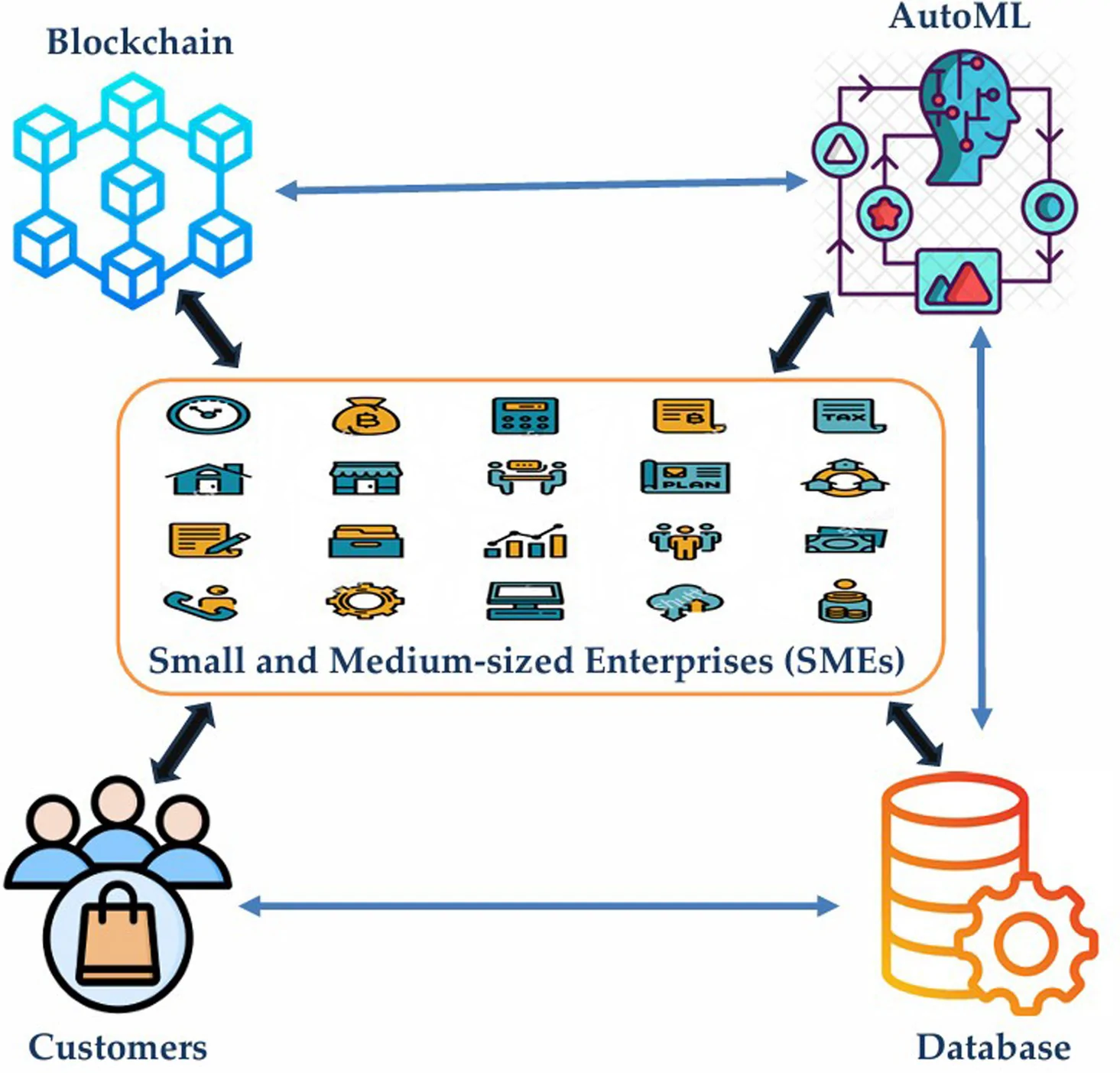
The proposed blockchain-based AutoML for SMEs.

Figure 2 displays the framework of the proposed architecture, which is broken down into different layers. The SME network includes the database, customer, AutoML, and blockchain security layers. The consortium network, which other parties host, owns the blockchain layer.

1. Customer Layer: Customers vary across industries. For instance, consumers are clients in the commodities trading business, just as patients are in the hospital sector. The consumer provides initial customer service data, which is an essential resource for businesses. These data are gathered using IoT devices and crowd sensing the consumer’s location, the state of the goods, etc. Businesses use the information they get about their customers to provide services for them.
2. SME Organization Layer: The organization consists of many establishments that offer customer support. These include non-governmental organizations, hotels, malls, and both physical and virtual stores. Employees collaborate with other layers to gather client information, which they then store in their knowledge warehouses for further investigation.
3. Application Layer: This layer includes several programs that businesses use to aid their customer care. Systems for managing cases, client relationships, customer care, etc., are included. For example, a customer service platform offering direct customer care forms customer service robots. The maintenance of communication logs and customer-organization interactions falls within the purview of the customer relationship administration system. Stated differently, the application layer stores a great deal of customer service information and ability via multiple application platforms. Decision support systems and robots for customer service are also included to provide client services.
4. Data Masking Layer: This layer describes the data masking procedure. It supports testing and design by enabling data replication in a nonproduction context without showing sensitive information. Removing recognizable characteristics from sensitive data and insisting data anonymization reduces the possibility of personal data being leaked. Number and date variation, encryption, nulling out or elimination, replacement, and scrambling are the five techniques for data masking. For instance, the user’s birthdate can be chosen randomly from any range of 120 days, thereby hiding the person’s identity while supporting the original distribution. Consequently, the application layer information is anonymized before being sent to the computing layer for data exchange and machine learning.
5. Technology Layer: The two sections of the technology layer, Blockchain, and AutoML, are hosted by outside organizations like data analysis agencies. The goal is to get trained models along with the right parameter setup. It is an ongoing learning process where optimization is carried out in response to fresh data. The outcomes serve as input to the business so that it may continue to offer computerized customer support. The four components of AutoML are model selection, transfer learning, deep learning, and feature selection. Feature discovery looks to provide superior training data characteristics by reducing noise and information compression. It increases resilience and makes machine learning training and applications more accessible.

The AutoML modules offer a variety of machine learning techniques, including neural networks with deep layers and hyperparameter tuning. These techniques provide greater accuracy than conventional machine learning techniques. The module collaborates with model selection to choose the best analytic techniques. A well-chosen model can enhance the evaluation’s efficacy and efficiency. Transfer learning refers to a pretraining model that is applied again to a related task. It aids in lowering the amount of data and training time.

Another essential part of the proposed architecture is blockchain. It offers a trustworthy and safe exchange of customer service information. It keeps track of the organizations’ encrypted hash of their data addresses. To ease the trading of disguised customer data, it forms many technological modules, including consensus, membership service, smart contracts, transactions, safe execution, event distribution, and asymmetrical encryption. Specifically, the consensus module offers a crucial method for quickly certifying and validating blockchain transactions. The blockchain member authority’s management service is called the member service module. Data exchange between several organizations may be done automatically using innovative contract modules. Intelligent contracts include built-in transaction rules covering costs, requirements, features, and data consumption. Data on transactions between various organizations is stored in the blockchain via the transaction module. The event distribution module manages the blockchain’s decentralization, enabling the decentralized storage of all recorded data. This system can guarantee both the transparency of the transactions and a lower chance of data manipulation. Data security is guaranteed during data transfer via an asymmetrical encryption module.

1. Evaluation Layer: The term “evaluation layer” describes how trust values of different organizations rank the caliber of information and trained machine learning algorithms. After using the model and data, the organization can assess the quality of the usage. The assessment outcome must be recorded on the blockchain. It contributes to ensuring the caliber of training services and information.

### 3.1 Blockchain-based reputation model for customer services

We incorporate our anonymous reputation system blockchain in this section. Creating rating tokens and consumer/retailer registration are off-chain procedures that call for safe communication between the IDM, stores, and customers. On a public ledger L , on-chain actions such as review creation, confirmation, and consolidation occur. In the proposed system, we use a hybrid blockchain approach. SMEs use the PoS protocol in Ouroboros to function as stakeholders, and their reputations are tied to the stake. SMEs can only act as stakeholders if they have received approval from IDM. Customers take part in the blockchain network as users and are free to join. Customers may add ratings and totals to the reputation scores of shops by adding various transactions to the ledger. We use smart contracts for the following three reasons.

1. Building a consortium network using a PoS blockchain is better.
2. Unlike a proof-of-work blockchain, a proof-of-stake blockchain provides superior scalability and efficiency.
3. In the proposed system, the consensus mechanism makes committee member management achievable.

The later steps outline the operation of our anonymous reputation mechanism based on blockchain. These steps are designed to ensure the integrity and transparency of the reputation system, providing a clear and reliable way to assess the reputation of shops and SMEs.

#### 3.1.1 Genesis block generation

The IDM executes the System Configuration of the blockchain network, which also creates and provides the system parameters P. P is available to businesses and consumers through secure channels, such as transport safety. The IDM also defines TA, which represents the size of the anonymous set and the system’s degree of privacy. Retailers communicate with the IDM to set up their public keys. The IDM compiles every store’s worldwide reputation score into a global reputation board B. Customers receive anonymous identification keys σi by registering with the IDM. Retailers and customers alike can sign transactions using a pair of public and private keys by connecting to the blockchain network and obtaining their blockchain identities. Consumer blockchain identities remain unidentified, while retailer blockchain data is publicly linked to their identity. The reputation score is calculated as follows in Equation 1:

$$RS_{j}=g_{2}^{\sum s_{i,j}}$$ (1)

From this, we can note that the final aggregated score is calculated based on the exponential value of $g_{2}.$ All retailers and consumers can effectively calculate the aggregated score for review generation. The IDM establishes its global clock and divides it into equal-time interval epochs. Three phases characterize each epoch: (1) Collection, (2) Grouping, and (3) Disclosure. For every stage, there are K1, K2, and K3 time slots available. The IDM chooses a committee of retailers with good reputation ratings using a committee selection mechanism at the start of each epoch. This committee oversees the choice of slot leaders and the disclosure process. The IDM then generates the system characteristics P, retailer variables Pj, retailer blockchain data, and the list of the committee (LC) members for this epoch into a genesis block of ledger L. Committee members conduct a leader selection function to choose slot leaders for periods in this epoch (Zhang et al., 2022).

#### 3.1.2 Review accumulation

The IDM generates an evaluation smart contract *SCj* for every enrolled retailer, *Rj*. For store *Rj*, reviews are recorded using the smart contract *SCj*. Specifically, the two methods, Update and *GetReview*, are part of the *SCj* agreement. The Update function considers customer anonymous review activities. The *GetReview* method then provides access to anonymous reviews. Consumer Ci can get a legitimate rating token *σi,j* by making off-chain transactions from merchant Rj. The legitimate rating token is obtained from the following Equation 2:

$$\sigma_{i,j}=\text{SigCom}(\mathrm{Ci},\mathrm{Pj},\mathrm{Sj},\mathrm{Rj})$$ (2)

The customer has access to public/private key pairs and a one-time blockchain address. Using the Update function, Ci may now create an anonymous review transaction Tr that includes the anonymous review ( σ,ri,j,πi,j,Rj ) for the intelligent contract *SCj*. The smart contract *SCj* stores anonymous reviews for eventual reputation consolidation and disclosure.

#### 3.1.3 Review aggregation

Each slot leader oversees reviewing the aggregation task of 1/K2 of all merchants throughout the Aggregation stage. In the later phases, slot leaders compile the encrypted reviews for every merchant.

1. A slot leader inquiry about the status of contracts within her management purview, *SCj*. To find the unruly customer’s real identity, the slot leader gives duplicate evaluations for the same store to the IDM.
2. The slot leader’s responsibility is to meticulously verify the quantity of legitimate evaluations that retailer Rj has received. Their careful addition of the legitimate encrypted rating scores is what leads to the aggregated rating score sj , especially if the total exceeds TA.
3. The slot leader builds R, a reveal smart contract with a counter CRj that keeps track of the retailer’s review scores. Contract R holds the total rating scores for the stores within her management purview.

Additionally, the smart contract, a key part of the process, offers the *Update-Token* method. This method, which allows committee members to give tokens for partial decryption, is a significant advancement in the system. The process moves forward to the last Revelation step after each of the slot leaders in this phase has published the R contracts.

#### 3.1.4 Review revelation

Members of the committee initially review the reveal contracts, or R, created in the earlier step during the disclosure stage. Committee members use the Update-Token method to add incomplete decryption certificates to the reveal contracts for the collected evaluation scores. Following the acquisition of every partial decryption token for each reveal contract, the IDM decodes the aggregated scores and confirms that the tokens are correct. Lastly, IDM changes the shops’ rating on the worldwide reputation board.

#### 3.1.5 Epoch update

Retailers communicate with the IDM to create a fresh set of retailer public keys, *Pj*, for every retailer *Rj* for the upcoming epoch. The IDM manages the committee choice procedure for the next period. Then, new committee members choose the leaders for this epoch using the most recent worldwide reputation ratings. Consumers produce new review transactions with the modified committee cryptography parameters for the classified reviews not aggregated in the earlier epoch.

### 3.2 Data flow of the proposed platform

**Figure 3:**
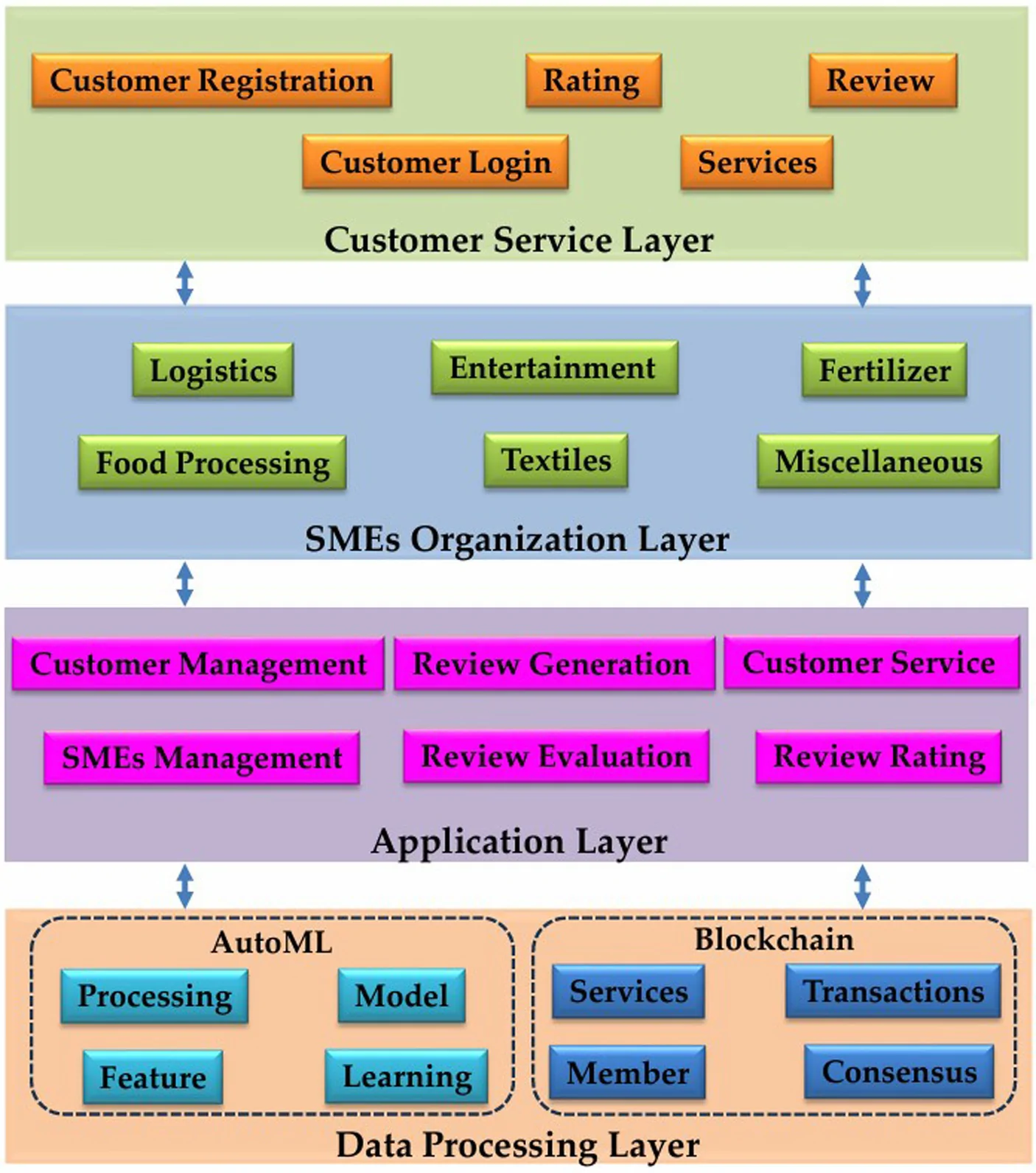
The proposed data flow model of blockchain-integrated AutoML for customer services.

The smart contract used in this study was coded in Solidity, a popular language for blockchain implementation. Its goal is to enable automated data and service trade. Figure 3 depicts the proposed platform’s data flow. An organization must choose an AutoML system and data provider and execute smart contracts during the smart contract execution step. The company looks through the market and chooses AutoML service and data providers. The trading system verifies the accuracy of the information by cross-referencing the rating with the reputation model. After that, the company initializes smart contracts using them. The disguised data address of the data firm is created into a hash. The other parties confirm the smart contracts when recorded on the blockchain. Upon confirmation of the contract, the organization receives the masked data from the data business access and the authority to gain access to the related AutoML platform. The smart contract’s access control part will carry out the procedure of providing access once the deal and payments have been confirmed. At that point, the contract cannot be changed. The AutoML platform receives masked data from the organization throughout the learning phase. The platform then uses this data to train and returns the learned model and its relevant parameters. The blockchain-integrated reputation-based AutoML frameworks allows different SMEs to securely exchange the customer reviews, automatically select ML model, and implement the model through smart contracts without need of centralized server.

**Figure 4:**
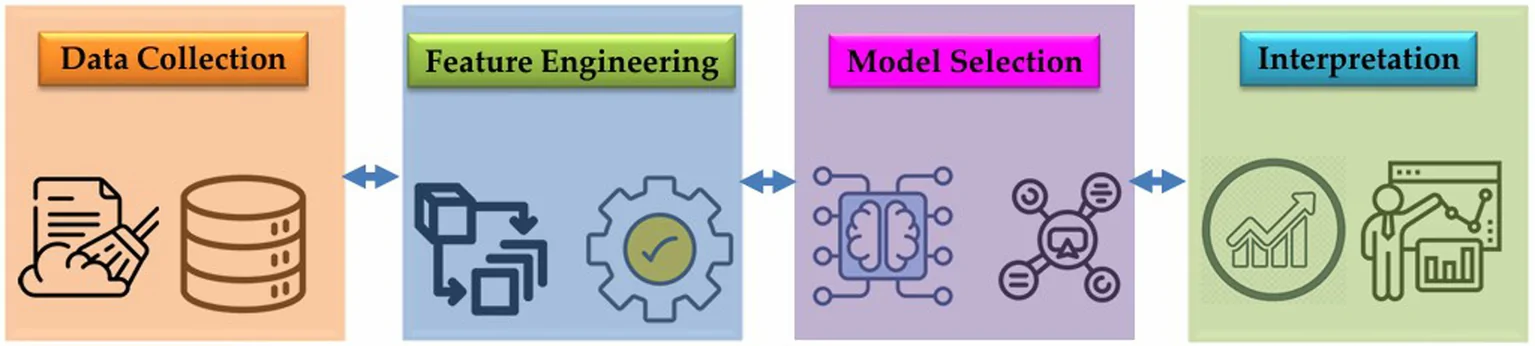
The proposed AutoML framework for customer services.

The framework of AutoML is shown in Figure 4. Every stage, including data reading, pre-processing, model generation, and result prediction/interpretation, is managed and carried out by AutoML framework. In the proposed framework the data collection and interpretation are the two primary areas of concentration for AutoML. It is simple to automate any further intermediate stages. Furthermore, AutoML offers best models that are prepared for forecasting. Based on scikit-learn, Auto-SKLearn is an automated machine learning that we have integrated with blockchain for customer review. A machine learning user is released from algorithm selection and hyperparameter adjustment by Auto-SKLearn (Feurer et al., 2020). It covers feature engineering techniques including PCA, feature selection, and hyperparameter tuning. The AutoML model handles regression and classification tasks using SKLearn estimators. To refine the channel, Auto-SKLearn builds a pipeline and applies Bayes search to improve the learning performance. Two elements are added to the ML framework to use Bayesian reasoning for hyperparameter tuning: During the optimization phase, meta learning is used to assess the auto-collection building of the setup and populate optimization algorithms using Bayes. An AutoML effectiveness depends on the hyperparameters being configured correctly. Therefore, hyperparameter adjustment is needed throughout this learning to enhance the model’s efficacy on the customer review dataset. It is important to note that both integer and category variables are included in the entire values of dataset space for hyperparameter optimization. We also applied Bayesian optimization since it employs a Gaussian method that takes prior parameter tuning into account, while grid search does not. Furthermore, grid search is inefficient and prone to dimensionality explosion when there are numerous parameters, while Bayesian conditioning is quick and requires fewer repetitions.

AutoML trains and assesses machine learning algorithms using the gathered data to find patterns and qualities in the data. This approach outsources the training of machine learning designs, as opposed to the traditional practice of directly exporting customer service to outside companies. The firm’s archives hold the fundamental outcomes and customer service procedures. In the application step, the trained model is after used. An assessment process is suggested to set up a trade ecology that is morally upright. Organizations can rate the AutoML platform and buy data throughout the assessment process. It supports the sector in keeping ambitious standards for information and services. The evaluation’s findings are likewise recorded in blockchain as an unchangeable seller’s reputation history. Both empirical and nonquantitative indices are part of the data assessment indexes. Customers can ask the service robot or resort staff member about nearby organizations if the hotel uses the system and wants to know. To aid the robot and front desk, the back-end customer care system will ask for added details from the consumer, such as their age, gender, and location, and will then offer relevant feedback. AutoML is used to train the system’s predictive model, and the data it uses comes from many connected hotels or businesses. Blockchain is used for storing data exchange. The comments will also be added back into the software as a fresh record for the SMEs to use once the client gets a response from the front desk.

## 4 Analysis of the proposed framework

To assess the suggested platform, the time consumption and experimental tests are conducted on a Windows 10 PC with an Intel Core i5 CPU 1.50 GHz and 8 GB of RAM. As previously stated, our experiments run on a private Ethereum chain. The private network is configured using Ganache, and its parameters are modifiable (Zheng et al., 2022). This method allows a high degree of parallelism and aids with segment memory hardening. It resolves all known vulnerabilities, including hash interoperability attacks, parallel queries, pseudorandom arrays, memory saving, and precomputation attacks. Lastly, the suggested platform was evaluated and debugged using the ETH test net: the security and proposed architecture compared with other centralized and decentralized network models presented in Table 1.

**Table 1:** Proposed architecture overview.

Reference framework	Network model	Anonymity	Privacy	Transparency	Confined separation
Soska et al. (2016)	Centralized	Yes	Yes	No	Yes
Hoyer and van Straaten (2022)	Decentralized	Yes	Yes	No	Yes
Yang et al. (2023)	Decentralized	No	Yes	Yes	No
Li et al. (2021)	Decentralized	Yes	No	Yes	Yes
Nguyen and Simkin (2017)	Decentralized	Yes	No	Yes	Yes
Proposed	Decentralized	Yes	Yes	Yes	Yes

### 4.1 Smart contract cost and time consumption

After measuring the gas and time consumption of the smart contracts, the distributed ledger and smart contracts based on the ETH platform are constructed to assess the operating cost of the suggested platform. Gas is one resource that must be used while smart contracts are being executed (Van Leeuwen et al., 2020). Various operations need various amounts of gas. The value of petrol consumption increases with operating complexity. As a result, the amount of gas used during an intelligent contract operation stands for the complexity of the innovative contract procedure. On ETH, a token called “Gas” may be traded at a specific ratio. As a result, calculating gas consumption is like estimating the cost of executing a smart contract. The primary features of this platform’s smart contracts were chosen, and their time and gas consumption was calculated, as Table 2 proves.

**Table 2:** Time consumption of different entity registration.

Process	Entities	Time consumption (ms)	Gas used (wei)
SMEs registration	SMEs ID	234	1,897,900
Consumer registration	Consumer ID	432	1,685,700
Review rating	Reputation model	243	2,356,800

### 4.2 Blockchain network evaluation and security analysis

**Figure 5:**
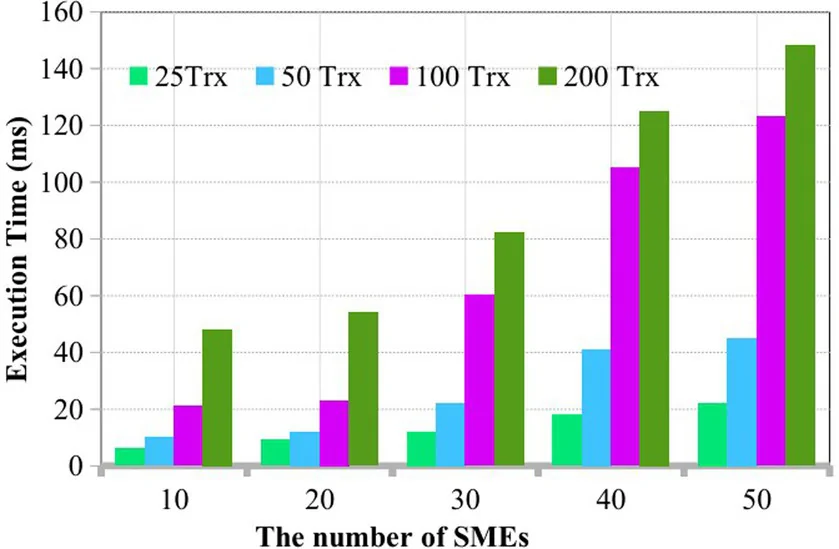
The block creation time for different file transactions.

**Figure 6:**
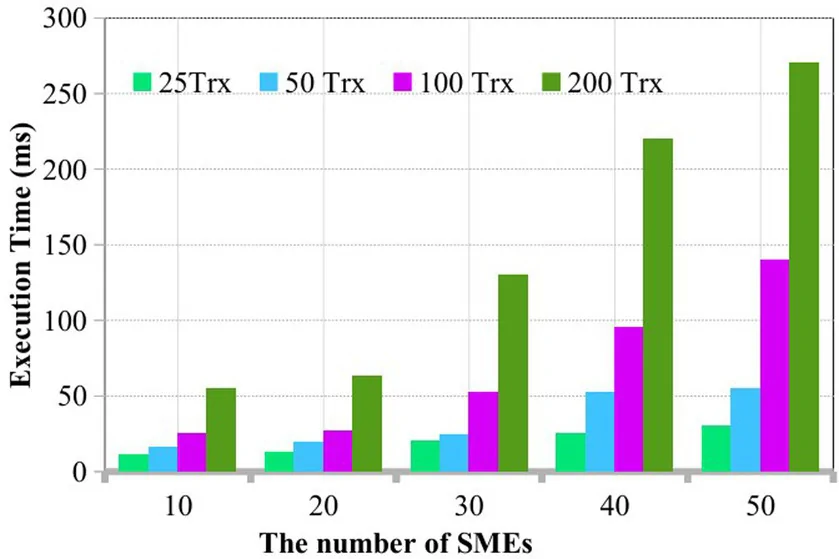
The block access time for different file transactions.

**Figure 7:**
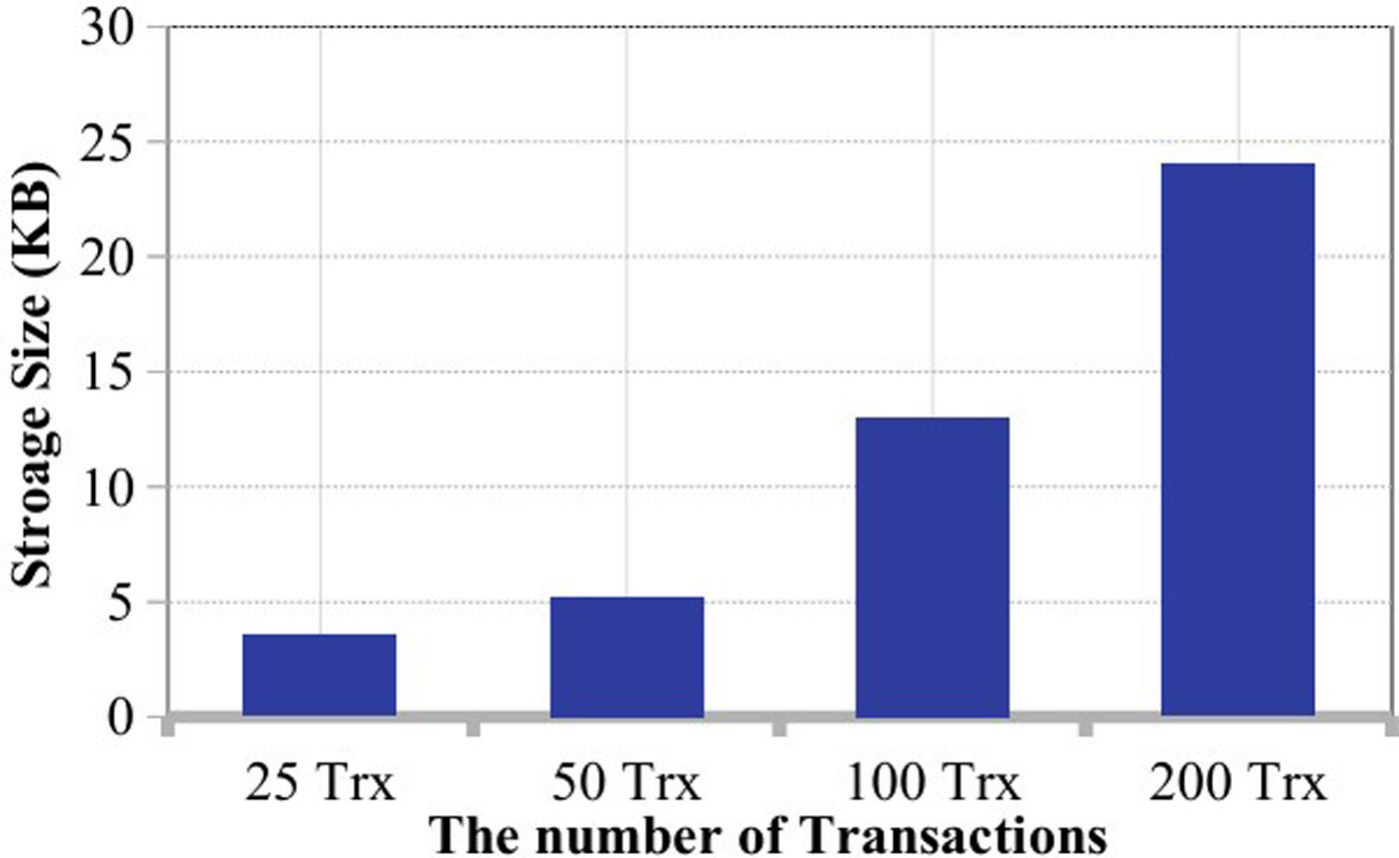
The blockchain network storage size for different file transactions.

Three primary indicators are described for blockchain network evaluation, and the suggested network is contrasted with current public networks. Every SME organization in the proposed design must be verified to guard against malicious activity and guarantee security and privacy. We have shown the time needed for block creation and block access intervals in Figures 5, 6. It is clear that, as would be expected, time rises as the number of SMEs grows. The actual storage capacity in KB for various transaction counts within the framework is displayed in Figure 7. IDM secure and distributed storage layer is used to calculate the storage sizes. The purpose of the off-chain activities is to increase the scalability of the structure. As the number of transactions rises, so does the storage size (measured in KB).

In this section, we provide a security analysis of the blockchain network for AutoML-based customer services in SMEs.

#### 4.2.1 Sybil attacks

The decentralized IDM partitions a single organizational entity into multiple pseudonymous verification nodes to secure inconsistent stake in the network. This will be eliminated by SME registration and authorized by the entire decentralized network.

#### 4.2.2 Collusion attacks

Malicious nodes coordinate to form alliances, unnaturally raise ratings, control reliable reviews, or perform 51% attacks. To prevent collusion attacks, we have proposed a reputation system consisting of several layers and an AutoML framework-based blockchain network to mitigate 51% attacks.

#### 4.2.3 Malicious review detection

Enemies propose fake, spam, or review-bomb inputs to interrupt platform functionality or perform “whitewashing.” By implementing reputation score-based review generation and verification through a blockchain network, fake reviews in the system can be avoided.

#### 4.2.4 Privacy guarantees

In the proposed system, both customer service data and SMEs are stored in the local network, and transaction details are stored in the blockchain network. Thus, there is no possibility of leaking privacy information for both customer and SME data.

The above-mentioned security analysis proves that the proposed blockchain-integrated AutoML framework is highly suitable for SMEs.

### 4.3 AutoML evaluation

The dataset used in this study, sourced from Kaggle’s E-commerce Customer Behavior [450 rows and 3 KB], is derived from real-world data collected from various online platforms. These datasets reflect actual transactions, customer behaviors, and common marketing performance metrics. Using these pre-existing datasets, the study can assess the effectiveness of proposed blockchain-integrated AutoML models for automated customer services. This approach ensures that the study remains practical and grounded in real-world data scenarios while highlighting the impact of automated data-driven decision-making for SMEs.

**Figure 8:**
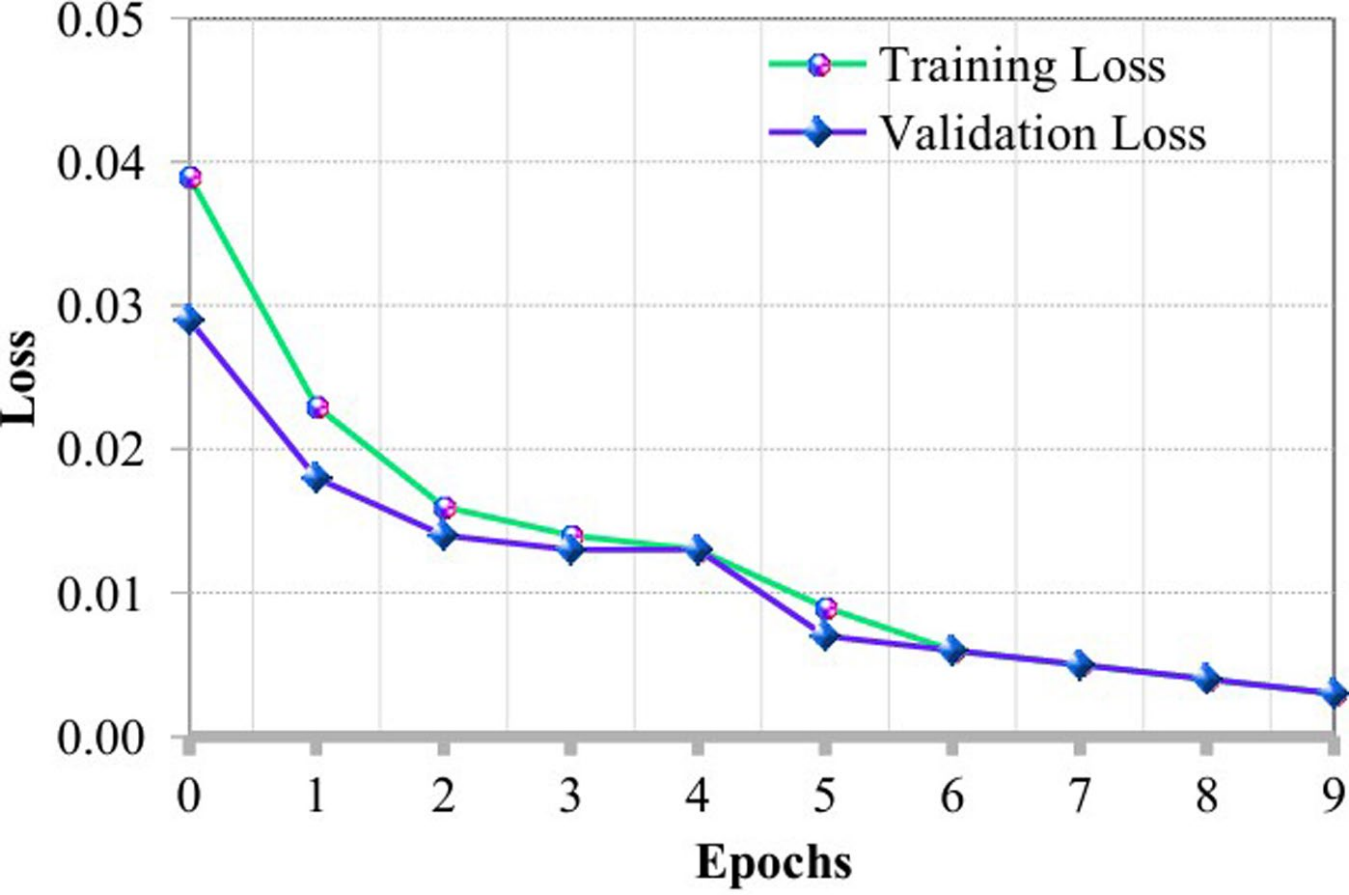
AutoML model training and validation loss.

**Figure 9:**
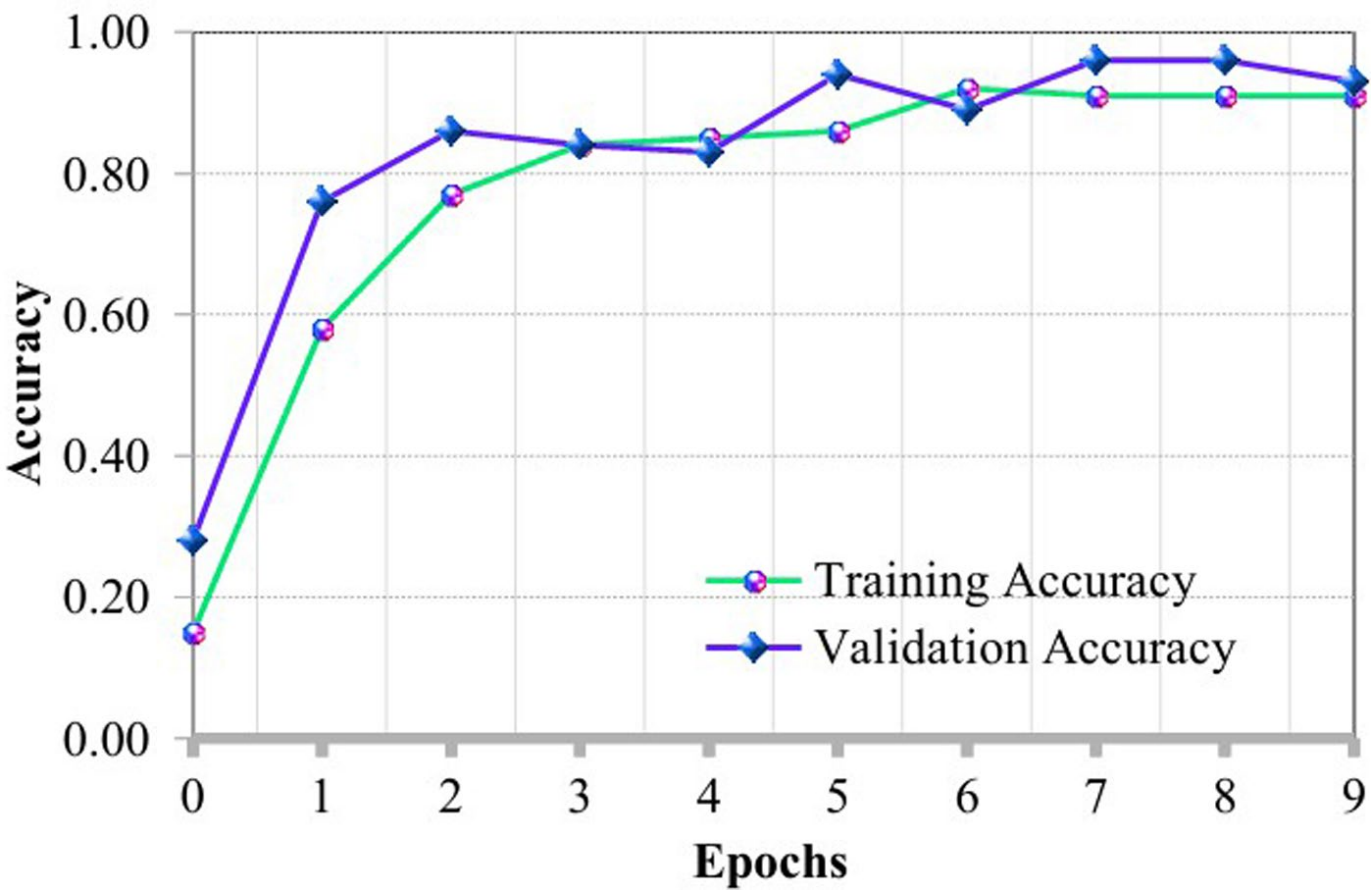
AutoML model training and validation accuracy.

This section examines the effects of the proposed AutoML strategy, followed by an analysis of the performance of automated customer services. We considered hyperparameters selected for customer datasets based on trial-and-error studies. The blockchain architecture enables AutoML to be well-designed for managing customer rating data. The accuracy vs. loss obtained from the blockchain technique with AutoML is shown in Figures 8, 9. The proposed approach has demonstrated an efficient ability to learn from customer datasets, yielding around 95.53% validation accuracy and 0.0059% loss, and 82.60% training accuracy and 0.0149% loss. The proposed framework encodes the provided datasets into a new structure using the blockchain approach, which can prevent inference attacks. AutoML would then use these encoded datasets to find and categorize attack activities.

In this work, we examined AutoML’s effectiveness in connection to a customer review in an SMEs case study, particularly regarding anonymous customer service. We contrasted AutoML’s performance with matching outcomes from traditional logistic regression (Jain et al., 2020) and neural networks (Thangeda et al., 2024). We discovered that the AutoML algorithm performs better based on our training data, as described in Table 3. However, the comparatively straightforward logistic regression produces marginally less performance on the independent test data, particularly in terms of accuracy and F1 score. The fact that our database only has a large number of features may be one reason why sophisticated models, such as the neural network and the AutoML method, could outperform logistic regression. Therefore, there is undoubtedly potential for obtaining even more discriminatory power using more sophisticated algorithms than logistic regression, such as AutoML. Finding a proper mix of feature selection and processing, model selection, and hyperparameter tuning is crucial for achieving high discriminatory power with an AutoML method. This process can easily become quite difficult and computationally demanding because of the various aspects involved.

**Table 3:** Classification results and performance comparison of proposed AutoML with existing baseline neural network and logistic regression models.

Performance metrics	AutoML	Neural network	Logistic regression
AUC	0.836	0.819	0.796
Accuracy	0.846	0.816	0.789
F1 score	0.853	0.845	0.839
Precision	0.839	0.817	0.823
Hyperparameter tuning	Yes	No	No

Furthermore, logistic regression models are often simple to understand. For example, our logistic model with only three variables is easy to understand. These algorithms still have a certain black-box quality. Additionally, neural networks often exhibit a particular “black-box” feature. Neural networks also have the drawback of often requiring a lot of data to train the algorithm and provide applicable findings. This may be linked to equally lengthy computation durations. Nonetheless, neural networks often develop effective solutions, even for challenging and highly nonlinear issues. AutoML has several benefits over neural networks and other machine learning techniques. First, it should be noted that even sophisticated models are easy to create. Numerous settings may be selected, such as the methods to be evaluated or the duration of the simulation. AutoML offers a simple method for finding the types of algorithms that may be especially discriminated against and require further investigation. For customer review datasets, automated machine learning is a particularly promising tool.

### 4.4 Review system performance

**Figure 10:**
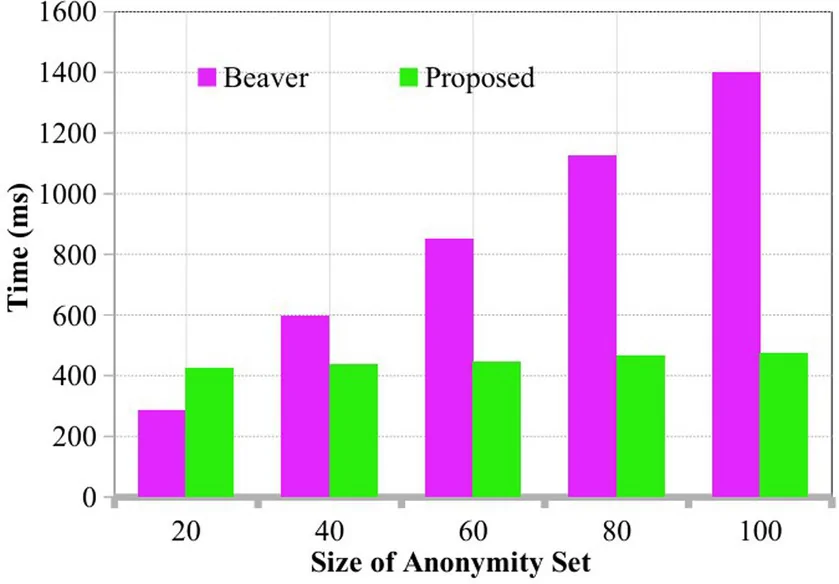
The time consumption of review creation.

**Figure 11:**
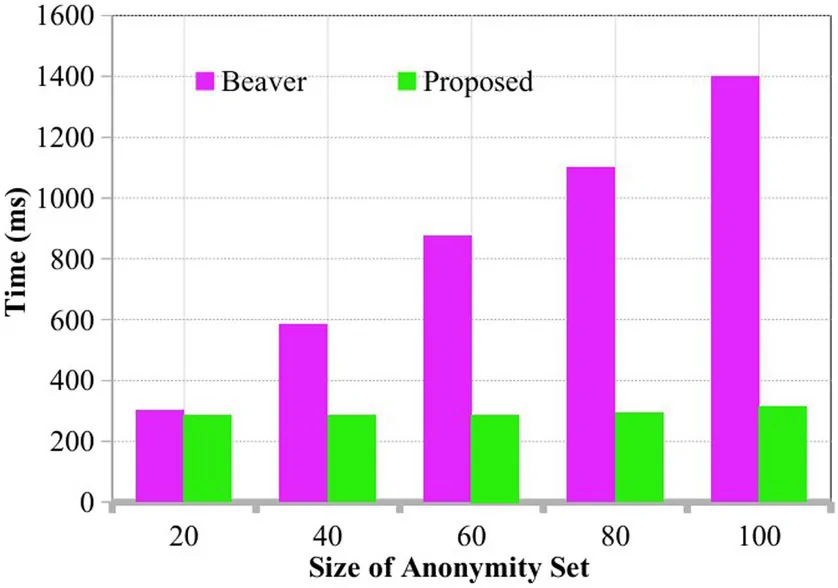
The time consumption of review verification.

We assess the system’s effectiveness, including the creation of review tokens among SMEs and customer registration. The findings from the simulation in Table 2 show that the processing time takes a few milliseconds. Customers with identification keys and rating tokens use the Update method in the review agreement to send anonymous reviews. Next, the slot leader obtains every review from the review contracts and confirms that the proofs are correct. To get partial decoding tokens from blocks, the slot leader sets up a disclosed contract R that combines the encoded evaluation scores of legitimate reviews. We also compare the proposed and the most recent research on review creation and verification based on ring identity (Soska et al., 2016). It is also necessary to post transaction activities on the public ledger using a ring-signature-driven technique. To create/verify the anonymous evaluations, customers gather a set of public keys of prior expenditures. This process results in a linearly rising computing cost, as seen in Figures 10, 11. The review creation and verification in the proposed architecture might take a few hundred milliseconds.

The following sections cover the system’s adaptability to various phases within a single epoch. The number of committee members during the period is defined as NC.

1. Accumulation Stage: If a user runs the Update method on our test PoS blockchain under ideal network circumstances, the customer can review transactions in the ledger in a matter of blocks. However, a transaction may be excluded from the epoch in real-world deployments due to delays in communication among consumers and slot holders. We have potential solutions in place. We may boost the total number of peer connections for the customer’s integrity node and the number of slots K1 in this phase to help alleviate this issue.
2. Aggregation Stage: During this phase, slot leaders confirm and compile the anonymous evaluations. The size of the anonymous set TA and the number of time slots K2 have a significant impact on performance. A higher K2 lengthens the overall epoch period but decreases the particular processing cost for slot leaders. The value of TA reflects customer privacy guarantees, a key feature of our system. A higher TA, however, may also raise the likelihood that an insufficient number of reviews are received for aggregation in this epoch, forcing users to resubmit their reviews in the following one.
3. Revelation Stage: The committee participants send coins to the exposed contract for partial decryption. NC∗K2 is the total number of operations in this phase. IDM can select several NCs to balance safety, effectiveness, and efficiency. The threshold of the ElGamal encryption method may increase, further revealing effectiveness, and avoid decryption failure if someone on the committee does not update the decryption token. The committee might potentially be divided into many subgroups. To cut down on communication overhead, each subgroup oversees managing a set of merchants’ decryption keys.

## 5 Conclusion

Conventional customer service depends on internal company capabilities. This study proposes a blockchain and AutoML method that combines the automated benefits of AutoML with the automated and decentralized benefits of blockchain. In contrast to conventional customer service techniques, the suggested platform creates a collaborative and reliable environment for information exchange. It is especially beneficial for SMEs to gather enough data to achieve automatic customer services and strengthen their core customer service competency. This paper provides a framework for SMEs to use shared data to enhance their customer services and reputation. The proposed architecture also expands to facilitate business collaboration for cooperative customer services. Using blockchain, the proposed system allows several customer care systems to access the same client data, ensuring service continuity and privacy. The blockchain’s interaction with AutoML enhances the security and traceability of SMEs data. The proposed reputation systems are implemented with a blockchain network to verify the trustworthiness of customer reviews and SMEs. The proposed architecture was confirmed with different experimental analyses. The experimental results show that the proposed architecture performs better than existing models and automates customer services. The AutoML model efficiency loss is calculated to measure the performance of the test and training models. The experimental results of AutoML provide acceptable efficiency and error for SMEs customer services. The proposed architecture is most suitable for decentralized SMEs customer services.

## Data Availability

The original contributions presented in the study are included in the article/supplementary material, further inquiries can be directed to the corresponding author/s.
